# *Candida albicans*-Epithelial Interactions: Dissecting the Roles of Active Penetration, Induced Endocytosis and Host Factors on the Infection Process

**DOI:** 10.1371/journal.pone.0036952

**Published:** 2012-05-14

**Authors:** Betty Wächtler, Francesco Citiulo, Nadja Jablonowski, Stephanie Förster, Frederic Dalle, Martin Schaller, Duncan Wilson, Bernhard Hube

**Affiliations:** 1 Department of Microbial Pathogenicity Mechanisms, Leibniz Institute for Natural Product Research and Infection Biology – Hans Knoell Institute Jena (HKI), Jena, Germany; 2 Laboratoire Interaction Muqueuses-Agents transmissibles (EA 562), IFR Santé-STIC, Université de Bourgogne, Faculté de Médecine, Dijon, France; 3 University Hospital, Dijon, France; 4 Department of Dermatology, Eberhard-Karls-University Tübingen, Germany; 5 Friedrich Schiller University, Jena, Germany; 6 Center of Sepsis Control and Care, Jena, Germany; University of Minnesota, United States of America

## Abstract

*Candida albicans* frequently causes superficial infections by invading and damaging epithelial cells, but may also cause systemic infections by penetrating through epithelial barriers. *C. albicans* is a remarkable pathogen because it can invade epithelial cells via two distinct mechanisms: induced endocytosis, analogous to facultative intracellular enteropathogenic bacteria, and active penetration, similar to plant pathogenic fungi. Here we investigated the contributions of the two invasion routes of *C. albicans* to epithelial invasion. Using selective cellular inhibition approaches and differential fluorescence microscopy, we demonstrate that induced endocytosis contributes considerably to the early time points of invasion, while active penetration represents the dominant epithelial invasion route. Although induced endocytosis depends mainly on Als3-E–cadherin interactions, we observed E–cadherin independent induced endocytosis. Finally, we provide evidence of a protective role for serum factors in oral infection: human serum strongly inhibited *C. albicans* adhesion to, invasion and damage of oral epithelial cells.

## Introduction

*C. albicans* is a normal member of the human microbiota and colonizes the oral cavity, gastrointestinal tract and urinogenitary tract of up to 70% or more of the population [1], [2], [3]. However, under certain conditions, the fungus can cause superficial infections at these different mucosa, such as oropharyngeal candidiasis (OPC) or vulvovaginal candidiasis (VVC). Moreover, if *C. albicans* disseminates through these epithelial barriers and reaches the bloodstream, it can cause life-threatening systemic infections. Therefore, *C. albicans* is adapted to interact with a wide range of host cells and molecules, both during commensal colonization and various disease manifestations. During both commensal and pathogenic relationships, attachment to the epithelial cells represents the initial stage of interaction [4], [5], [6]. Such attachment events are primarily mediated by adhesins on the fungal cell surface, which can interact with secreted factors, such as extracellular matrix (ECM) or serum proteins, immobilized ligands, such as integrins or cadherins, or indirectly, via other microorganisms. In contrast to microbial adhesion, invasion and damage of epithelial cells are largely considered to be solely pathogenic, rather than commensal attributes [7]. *C. albicans* can use two distinct invasion mechanisms to gain entry into host cells: induced endocytosis and active penetration [8], [9]. Invasion via induced endocytosis is dependent on dynamic microfilaments of the host, whereas active penetration relies on fungal viability. Phan et al., (2007) demonstrated that passive uptake of *C. albicans* by epithelial cells is driven by the interaction of the GPI-anchored hypha-associated protein Als3 with mammalian cadherins. This interaction mimics the formation of adherence junctions, leading to actin cytoskeleton rearrangements and subsequent internalization of the fungal cells [10]. Recently, a second *C. albicans* invasin, the heat shock protein 1 (Ssa1), was identified, demonstrating that *C. albicans* uses more than one invasin to induce endocytosis [11]. However, we have previously demonstrated that *C. albicans* cells are not endocytosed by enterocytes [9], indicating that epithelial cells also differ in their response to the fungus [9], [12]. Invasion into epithelial cells via active penetration presumably relies on a combination of physical pressure exerted by the extending hypha, the secretion of hydrolytic enzymes and as yet unknown damaging factors. However, the exact mechanisms underlying active penetration are largely unknown.

Host cell invasion can also be facilitated by binding extracellular matrix or serum proteins as bridging molecules – a strategy described for several bacterial pathogens [13], [14], [15], [16]. *C. albicans* is able to bind human serum components such as Factor H [17] as well as the extracellular matrix proteins laminin, fibronectin, collagen, entactin, vitronectin and tenascin [4] and binding of vitronectin by the integrin-like vitronectin receptor has been shown to reduce yeast cell adhesion to human endothelial cells [18]; however, it remains unknown whether *C. albicans* can utilize extracellular components as bridging molecules to facilitate invasion. In this study, we evaluated the cellular mechanisms of *C. albicans* epithelial invasion to elucidate the relative contributions of active penetration and induced endocytosis. Furthermore, we investigated the effects of E-cadherin, soluble ECM molecules and human serum during interaction with oral epithelial cells. We provide novel evidence of an Als3-E-cadherin-independent endocytosis pathway, but demonstrate that active penetration is the dominant epithelial invasion mechanism. Significantly, invasion and damage of oral epithelial cells was found to be specifically inhibited by human - but not by bovine - serum, and this effect was independent from active complement components.

## Results

### Dissecting the Invasion Routes of *C. Albicans* Hyphae into Oral Epithelial Cells

In order to assess the relative contributions of the two known invasion mechanisms of *C. albicans* [9], we selectively blocked either induced endocytosis with the microfilament inhibitor cytochalasin D (cytD) or active penetration by killing the fungal cells with thimerosal (Thim). The remaining invasion potential in the presence of cytD is host actin-independent and predominantly driven by fungal activity (active penetration only). When thimerosal was used to kill *C. albicans* hyphae, the remaining invasion is solely driven by epithelial cell activity (induced endocytosis only). The effectiveness of this approach was investigated by analyzing the invasion potential of thimerosal-treated fungal cells into cytochalasin D-treated epithelial cells. With this approach, no epithelial invasion occurred within the 3 h time of the experiment, indicating that cytochalasin D fully blocked induced endocytosis within the course of the experiment and that at least one route must be available for fungal invasion into epithelial cells. Note that for determining induced endocytosis, killed *C. albicans* hyphae, rather than yeast cells, were used because yeast cells are not endocytosed by epithelial cells [9].

### Electron Microscopy of Induced Endocytosis and Active Penetration

**Figure 1:**
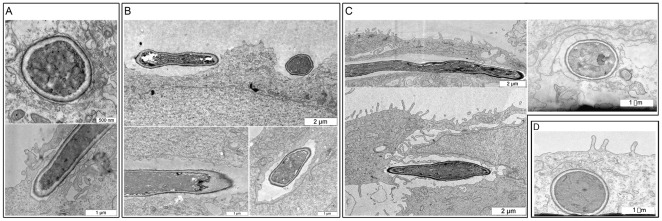
Transmission electron microscopy of *C. albicans* epithelial invasion. Induced endocytosis of thimerosal killed hyphae (A), invasion of cytochalasin D treated epithelial cells by viable hyphae (B), invasion of untreated epithelial cells by viable hyphae (C), invasion of inactivated epithelial cells by viable hyphae (D).

Previous scanning electron microscopy (SEM) studies of viable hyphae invading oral epithelial cells have demonstrated both depressions of epithelial cell surfaces (indicative of active penetration) and membrane ruffling and epithelial cell protrusions (indicative of induced endocytosis) [8], [9]. To further dissect the cellular events associated with these two invasion mechanisms, we employed transmission electron microscopy (TEM) of (1) oral epithelial cells co-incubated with thimerosal killed *C. albicans* hyphae (induced endocytosis only), (2) cytD treated epithelial cells co-incubated with viable *C. albicans* (active penetration only) and (3) untreated epithelial cells co-incubated with viable *C. albicans* (both invasion mechanisms). Thimerosal killed hyphae (induced endocytosis only) were engulfed by oral epithelial cells and tightly surrounded by the host membrane (Fig. 1A, upper panel). Membrane ruffling was visible around engulfed hyphae (Fig. 1A, lower panel). In contrast, oral epithelial cells treated with cytD (active penetration only) did not exhibit filopod formation or membrane ruffling (Fig. 1B). Invasion of hyphae occurred not only vertically (tip-first), but also laterally, with few direct physical contacts between hyphae and epithelial surface structures (Fig. 1B upper picture). Furthermore, invading hyphae were not tightly surrounded by host membranes in the presence of cytD. Rather we observed invaginations and broader spaces between penetrating hyphae and host membranes (Fig. 1B, lower pictures). Untreated oral epithelial cells invaded by viable hyphae (both invasion routes possible) generally reflected that of active penetration (invaginations and broader spaces between hyphae and host membranes). However, we also observed intermediate examples, with filopod-like structures and membrane ruffling on epithelial surfaces, but also the presence of broader spaces between hyphae and host membrane (Fig. 1C) similar to pictures shown previously [8]. We also tested the effect of complete epithelial inactivation on invasion by killing the epithelial cells with paraformaldehyde. In contrast to invasion of living cells by viable *C. albicans*, where we observed defined spaces between the fungal cell wall and the host membrane, invasion of killed epithelial cells was characterized by disrupted cellular structures around the invading hypha, with no evidence of an intact host membrane (Fig. 1D).

### Relative Contributions of Active Penetration, Induced Endocytosis and *C. Albicans* Invasins to Epithelial Invasion

We next sought to quantifiably evaluate the relative contributions of induced endocytosis and active penetration to *C. albicans* invasion. We therefore employed our selective inhibition approach and analyzed the invasion potential of *C. albicans* wild type and mutants lacking the two known fungal invasins, Als3 and Ssa1 [10], [11].

**Figure 2:**
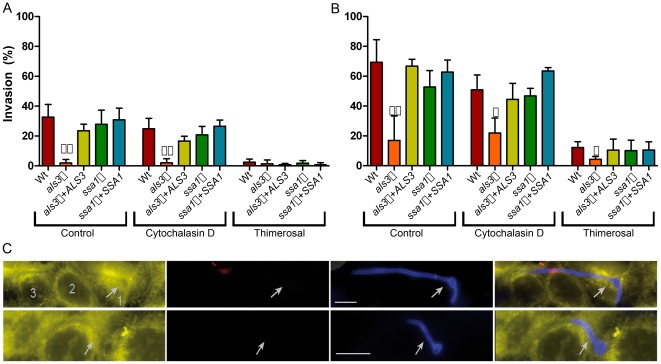
Contributions of fungal invasins, induced endocytosis and active penetration to *C. albicans* oral epithelial invasion. (A and B) Untreated or thimerosal-inactivated *C. albicans* cells were co-incubated with either untreated or cytochalasin D treated TR-146 oral epithelial cells for 2 h (A) and 3 h (B). After fixation, the samples were differentially stained and analyzed by fluorescence microscopy. The experiment was performed at least two times in duplicates. */**, indicates significant difference compared to the corresponding wild type control (Wt) (*p<*0.05/*p<*0.01, respectively). (C) Micrographs of invading hyphae. *C. albicans* appears blue (calcofluor white), with extracellular section of the hypha stained red (Concanavalin A); epithelial cells are stained yellow (Dil). Upper panel: a living hypha penetrating through several epithelial cells. Lower panel: a killed hypha, fully phagocytosed by an epithelial cell. Only viable hyphae are able to undergo inter-epithelial invasion. Numbers indicate the number of invaded epithelial cells and arrows mark internalized cells. Scale bar = 10 µm.

In agreement with previous studies [9], [19], killed hyphae (induced endocytosis only) were internalized by oral epithelial cells in a time dependent manner (Wt, Thimerosal, Fig. 2A and B). Occasionally, we observed both viable and killed hyphae which had been entirely internalized (Fig. 2C), demonstrating that induced endocytosis can result in complete uptake of *C. albicans*. Indeed, a noteworthy proportion of killed hyphae were internalized (2.5 and 12.2% at 2 and 3 h, respectively – Fig. 2A and B Wt, Thimerosal); however, the comparative invasion potential of untreated living fungi was much higher (32.7 and 69.4% at 2 and 3 h, respectively – control, Fig. 2A and B). As described previously, we did not observe internalization of killed yeast cells; these cells remained on the epithelial surface ([9] and data not shown).

Blocking induced endocytosis via cytD treatment (Wt, cytochalasin D, Fig. 2A and B) also reduced the invasion potential of *C. albicans*; however, by 2 and 3 h (24.8 and 50.8%, invasion respectively) the invasion inhibition elicited by blocking induced endocytosis was not as striking as for blocking active penetration. Together, these data demonstrate that *C. albicans* relies on both induced endocytosis and active penetration routes for optimal invasion during the early stages of interaction with epithelial cells, but that by 3 h, active penetration represents the dominant invasion mechanism.

Following epithelial invasion, viable *C. albicans* hyphae rarely remained in the primarily invaded cell, but rather continued to penetrate through the basal or lateral membranes and into the next neighbouring cell (Fig. 2C, upper panel). Typically, within 3 h, viable *C. albicans* hyphae penetrated from the primarily invaded cell and through several adjacent epithelial cells or exited the invaded cell and migrated through the extracellular space before invading subsequent cells (not shown).

Induced endocytosis of *C. albicans* has been shown to be mediated by Als3- and Ssa1- E-cadherin interactions [10], [11]. We therefore sought to quantitatively determine the contribution of both Als3 and Ssa1 to the two invasion routes. Viable cells or killed hyphae of *als3*Δ, *als3*Δ+*ALS3*, *ssa1*Δ and *ssa1*Δ+*SSA1* strains (kindly provided by Scott Filler) were used to infect oral epithelial cells and invasion kinetics monitored. At both investigated time-points, invasion of untreated epithelial monolayers by viable *als3*Δ was significantly reduced compared to the wild type under the same conditions and invasive potential was restored in the *als3Δ*+*ALS3* strain. This confirms previous studies that Als3 is important for invasion [10]. Deletion of *SSA1* also reduced *C. albicans* invasion into epithelial cells (24% less invasion than wild type), but this difference was not statistically significant.

At 2 h and 3 h post-infection, the invasion potential of *als3Δ* cells into untreated and cytD treated monolayers was virtually identical (*als3Δ*, control vs. *als3Δ*, cytochalasin D; Fig. 2A and B). This result, that blocking induced endocytosis in the absence of Als3 had no further effect on fungal invasion, confirms previous findings that Als3 mediates induced endocytosis. However, because the invasion rate of *als3Δ* into cytD-treated monolayers was significantly lower than for wild type cells (Wt, cytochalasin D vs. *als3Δ*, cytochalasin D; Fig. 2A and B), Als3 likely also plays a role in active penetration, possibly via anchoring *C. albicans* to the epithelial substrate. In contrast, deletion of *SSA1* did not result in further reduction in invasion of cytD-treated epithelial monolayers.

Interestingly, under conditions which permit induced endocytosis only (thimerosal-treatment of hyphae – *als3Δ*, Thimerosal; Fig. 2 B), invasion of *als3Δ* was not completely blocked, although significantly lower than that of thimerosal-treated wild type cells at 3 h post-infection. This remaining, albeit low level, invasion potential of killed *als3Δ* hyphae provides evidence that *C. albicans* possesses other factors which mediate induced endocytosis. However, because killed *als3*Δ hyphae were internalized around 65% less than killed wild type hyphae, we conclude that Als3 is one of the major invasins of *C. albicans*.

Based on these observations, we conclude that Als3 plays major roles in both induced endocytosis and active penetration.

Since fungal-driven active penetration appeared to represent the dominant route of invasion into oral epithelial cells, we next investigated whether invasion requires viable host cells. Therefore, TR-146 oral epithelial cells were killed by fixing with paraformaldehyde and invasion of *C. albicans* was quantified by differential staining. Killing of host cells did not prevent invasion. However, after 3 h, we observed approximately 30% reduced invasion into inactivated epithelial cells as compared to viable cells (data not shown), indicating that viability of host cells and native host cell properties are not essential for, but enhance invasion.

### The Invasion Efficiency of *C. Albicans* into Host Cells is Epithelial Cell Type Dependent

Induced endocytosis of non-professional phagocytic host cells occurs predominantly via cell surface associated protein interactions (e.g. Als3-E-cadherin), whereas active penetration relies on a combination of physical forces and directed hyphal growth [12].

To further examine the relative importance of the two invasion mechanisms and to determine whether invasion is epithelial cell type-dependent, we next investigated the invasion potential of *C. albicans* interacting with HeLa epithelial cells as compared to TR–146 oral cells. HeLa cells can be invaded by *C. albicans* [20], but do not express E–cadherin on the cell surface [21]. We first confirmed the presence of E–cadherin on the surface of TR-146, but not HeLa cells after infection with *C. albicans* via immuno-fluorescent microscopy (data not shown).

**Figure 3:**
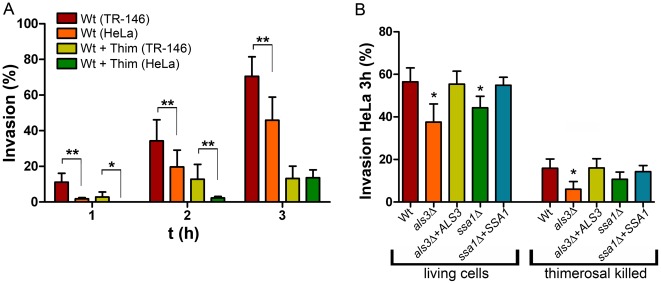
Differential **invasion of TR-146 or HeLa epithelial cells by** ***C. albicans*** **.** (A) Oral TR-146 or HeLa epithelial cells were co-incubated with 10^5^ *C. albicans* cells (alive or thimerosal killed – Wt+Thim) for either 1, 2 or 3 h. (B) HeLa epithelial cells were co-incubated with 10^5^ indicated *C. albicans* strains (alive or thimerosal killed) for 3 h. Following fixation, samples were differentially stained and analyzed by fluorescence microscopy. The experiment was performed at least two times in duplicates. */**, indicates significant difference between cell lines (A) or between mutant and wild type (B) (*p<*0.05/*p<*0.01, respectively).

We reasoned that if induced endocytosis was exclusively reliant on *C. albicans-* E–cadherin interactions on epithelial cells, then blocking active penetration should completely block invasion of HeLa cells. Indeed, during the first 2 h, killed hyphae invaded HeLa cells very poorly (Wt+Thim (HeLa), Fig. 3A). However, by 3 h, the number of killed hyphae endocytosed by TR-146 and HeLa cells was comparable. This data suggests the existence of a secondary, E-cadherin-independent endocytic pathway.

On the fungal side, Als3 and Ssa1 are required for E-cadherin-mediated induced endocytosis. Viable *als3Δ* exhibited significantly reduced HeLa invasion compared to the wild type and invasion was restored by *ALS3* complementation (Fig. 3B). However, the relative reduction in invasiveness of *als3Δ* into HeLa cells was not as strong as compared to TR-146 oral epithelial cells (Fig. 2A/B and Fig. 3B). Indeed, whilst wild type *C. albicans* invaded TR-146 more efficiently than HeLa cells, *als3Δ* cells exhibited a much greater defect in TR146 invasion. This reinforces the concept that Als3 plays a major role in E-cadherin-dependent epithelial invasion, but also functions independently of E-cadherin. Killed *als3Δ* cells (induced endocytosis only) were again internalized by HeLa cells, although to a lesser degree than the wild type and this defect was reversed in the *als3Δ*+*ALS3* strain. *ssa1Δ* also exhibited reduced invasion of HeLa cells. However, internalization of killed *ssa1Δ* cells by HeLa cells was comparable to the wild type.

Together these data suggest that, although Als3-E–cadherin interaction represents a major mechanism of induced endocytosis, other endocytic mechanisms exist, however, the specific mechanisms and receptors remain unknown.

Invasion potential is calculated based on the percentage of invading cells which remain attached to the epithelium following the differential staining procedure. However, the different treatments and genetic backgrounds analyzed may also influence the absolute number of cells which remain attached. We therefore determined the percentage of *C. albicans* cells which remained attached to the epithelium. Adhesion rates are summarized in Supplementary Table S1. Compared to living *C. albicans* cells, killed hyphae adhered poorly to both epithelial cell lines. Notably, even viable *als3Δ* cells adhered poorly to epithelial monolayers, indicating that the absolute (invasion events per inoculum) invasion defect of this strain is even greater than the “specific” invasion potential of attached cells.

### Host Factors Influence Adhesion, Invasion and Damage

Symptomatic stages of superficial *C. albicans* infections are characterized by damage and destruction of epithelial layers. Disruption of these barriers may result in increased exposure of extracellular matrix (ECM) molecules and the release of blood and blood components into the mucosal layer. Such extracellular components can be used by pathogenic microorganisms to facilitate infection or colonization of epithelial tissues by bridging between adhesins of the pathogen and epithelial receptors [15].

To investigate whether *C. albicans* can utilise ECM proteins during invasion, fungal cells were incubated with fibronectin, vitronectin, collagen, laminin or E–cadherin and both adhesion and induced endocytosis (invasion of thimerosal-killed hyphae) were independently assessed. Pre-incubation of either epithelial cells or *C. albicans* cells with fibronectin, vitronectin, laminin or collagen did not influence adhesion or induced endocytosis of *C. albicans* (not shown). On the other hand, pre-incubation of *C. albicans* with E–cadherin prior to killing significantly reduced fungal uptake by 48% (not shown), indicating that invasin molecule(s) on the surface of hyphae may have been blocked, thus preventing induced endocytosis.

**Figure 4:**
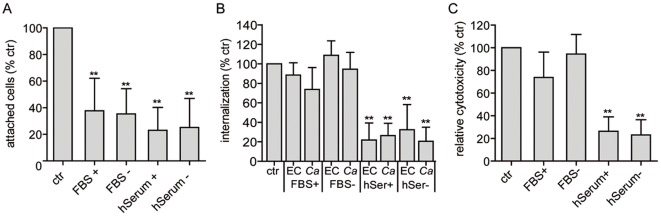
Adhesion, invasion and damage of oral epithelial cells by *C. albicans* in the presence of serum. (A) Adhesion of viable *C. albicans* cells to oral epithelial cells in cell culture medium supplemented with 10% human [hSerum] or fetal bovine serum [FBS]; (+) heat-treated; (−) untreated; ctr, *C. albicans* without serum. (B) Induced endocytosis of thimerosal-inactivated *C. albicans* hyphae. Either oral epithelial cells were supplemented with untreated and heat-treated 10% hSerum or FBS [EC] or *C. albicans* hyphae [*Ca*] were pre-incubated with untreated or heat-treated 40% serum; ctr killed hyphae without serum. (C) Cell damage of epithelial monolayers caused by viable *C. albicans* cells after 24 h in cell culture medium supplemented with untreated or heat-treated 10% hSerum or FBS compared to *C. albicans* infection with the addition of 1% serum (ctr). **, significant difference compared to control adhesion, invasion or damage (p≤0.01).

Next, we tested the influence of serum on *C. albicans* adhesion, invasion and damage. *C. albicans* was pre- and/or co-incubated with serum during interaction with epithelial cells. Both native or heat-treated (complement-inactivated) human or foetal bovine serum were used. Interestingly, despite having a positive effect on hyphal formation (not shown), treatment with either human or bovine serum strongly reduced adhesion to epithelial cells (Fig. 4A). Serum heat-treatment did not further affect adhesion, suggesting that complement, or other heat-labile components were not responsible for the observed serum-mediated inhibition of adhesion.

Invasion of thimerosal inactivated hyphae (induced endocytosis only) was not significantly affected by the presence of FBS (Fig. 4B). In contrast, induced endocytosis was dramatically and significantly reduced in the presence of human serum (Fig. 4C) and this effect was also observed upon pre-treatment of fungal cells. Heat-treatment of human serum did not further affect epithelial invasion, indicating that complement or other heat-labile components, do not influence the invasion process under these conditions. Finally, epithelial cell damage was strongly and significantly reduced when *C. albicans* was pre- and co-incubated with human, but not bovine serum (Fig. 4C). Again, heat-treatment did not influence the extent of epithelial damage. In summary, these data show that both bovine and human serum block *C. albicans* adhesion to epithelial cells, whilst human serum strongly reduced both epithelial invasion and damage.

## Discussion

### Cellular Dissection of the Early Stages of *C. Albicans*-epithelial Interaction

Microbial invasion of non-professional phagocytic host cells can occur via two general mechanisms: active penetration or induced endocytosis. Plant pathogenic fungi can actively penetrate plant cell walls by producing specialized structures (appressoria), hydrolases and high turgor pressures [22], [23]. Parasites, such as members of the apicomplexa group, can actively penetrate into host cells using actin-mediated forces [24]. Bacteria, on the other hand, cannot actively penetrate host cells, but have developed several strategies to cause uptake by induced endocytosis [25], [26]. Bridging molecules promote adhesion and can facilitate induced endocytosis [27]. Therefore, pathogenic microbes have developed different mechanisms for invading host cells.

*C. albicans* is capable of invading epithelial cells via both induced endocytosis and active penetration [8], [9], [10].

Endocytosis of *C. albicans* is induced by binding of fungal cell surface proteins such as Als3 and Ssa1 to host cadherins, followed by host clathrin, dynamin and cortactin accumulation at the site of internalization [11], [28]. The mechanisms by which *C. albicans* actively penetrates epithelial cells, on the other hand, has remained rather speculative, but presumed to rely on a combination of physical pressure exerted by directed hyphal growth, the extension of hypha and the secretion of hydrolytic enzymes [12].

The first aim of this study was to elucidate the relative contributions of these two invasion routes. To do this we: used two epithelial cell lines (TR-146, which express E–cadherin and HeLa, which do not); treated epithelial cells with cytD (to selectively block induced endocytosis); killed epithelial cells (to block all host activity); used *C. albicans* wild type or strains lacking the fungal invasins, Als3 and Ssa1; and killed the fungal cells with thimerosal (to selectively block active penetration). Together our data support a model whereby: *C. albicans* can invade via two distinct, yet complementary, mechanisms (induced endocytosis and active penetration); Als3-E–cadherin-mediated internalization represents the dominant mechanism driving induced endocytosis, but that an Als3- and E–cadherin- independent mechanism of induced endocytosis exists; finally, active penetration represents the dominant invasion route for *C. albicans* into epithelial cells.

### Induced Endocytosis is Mediated by Als3-E-cadherin- Dependent and Independent Mechanisms

Although Als3/Ssa1-E-cadherin binding on epithelial cells has been shown to mediate induced endocytosis [10], [11], we sought to determine whether E–cadherin independent endocytic pathways of epithelial cells exist. We therefore compared invasion rates of viable and killed wild type, *als3Δ* and *ssa1Δ* cells into TR-146 epithelial cells (either untreated or treated with cytD) or into HeLa cells. Our data confirmed that Als3 represents a major invasin of *C. albicans*; however, we also provide compelling evidence that an E–cadherin independent endocytic pathway exists.

Viable *als3Δ* exhibited significantly reduced invasion of both untreated TR-146 and HeLa cells. However, *ALS3* deletion had a greater negative impact on invasion into E-cadherin[^+^] TR-146 cells than into E-cadherin[^−^] HeLa cells, in agreement with previous studies that Als3-E–cadherin interaction contributes to epithelial induced endocytosis [10]. Moreover, blocking induced endocytosis in the absence of Als3 (via cytD treatment) did not further influence invasion, providing additional evidence that Als3 mediates host cytoskeleton reorganization. In addition, under conditions, which permit induced endocytosis only (killing of hyphae with thimerosal-treatment), uptake of *als3Δ* cells by TR-146 was 65% lower than *C. albicans* wild type cells. Together this confirms previous reports that Als3 represents a major *C. albicans* invasin, but that other invasins also trigger induced endocytosis of oral epithelial cells [10], [11]. These must include as yet undiscovered invasins, as deletion of the only other known *C. albicans* invasin gene, *SSA1* did not inhibit induced endocytosis by TR–146 or HeLa. It is possible that Ssa1-mediated endocytosis is cell type-specific, as the *ssa1Δ* mutant has previously been shown to exhibit impaired endocytosis of endothelial and FaDu epithelial cells [11].

Als3 would also appear to significantly contribute to invasion via active penetration, because invasion of *als3Δ* into cytD treated TR-146 cells (active penetration only) was significantly reduced compared to the wild type under the same conditions. Together with our data that *als3Δ* exhibited reduced invasion into HeLa cells (which do not express E-cadherin), this would suggest that Als3 contributes considerably to active penetration, possibly via its adhesin function, by anchoring the fungal cell to the epithelium.

On the host side, E–cadherin is the only epithelial surface receptor known to mediate endocytosis of *C. albicans* [10], [11]. In line with this, we observed virtually no internalization of thimerosal killed *C. albicans* hyphae (induced endocytosis only) into [E-cadherin^−^] HeLa cells within the first 2 h of co-incubation. Strikingly though, by 3 h, HeLa cells had internalized similar numbers of killed *C. albicans* hyphae as TR-146 cells. Because HeLa cells do not express E–cadherin ([21] and this study) this demonstrates that *C. albicans* can be endocytosed via an E-cadherin-independent endocytic pathway. It is likely that Als3 either alone or as part of a multiprotein complex contributes to this E-cadherin-independent endocytic mechanism, as killed *als3Δ* were endocytosed by HeLa cells less efficiently than the wild type.

### Active Penetration Represents the Dominant Invasion Route

One of the major aims of this study was to dissect the relative importance of induced endocytosis versus active penetration to epithelial invasion. Living *C. albicans* on untreated TR-146 epithelial monolayers, capable of invading via either induced endocytosis or active penetration, exhibited invasion rates of 32.7% and 69.4% at 2 h and 3 h, respectively. Selectively blocking induced endocytosis reduced the invasion potential to 24.8 and 50.8% at 2 and 3 h, respectively. Selectively blocking active penetration, on the other hand, reduced the invasion potential to 2.5 and 12.2% at 2 and 3 h, respectively. Therefore, within the first 3 h of infection, active penetration accounts for approximately 70% of *C. albicans* invasion potential. This is supported by the fact that killing epithelial cells resulted in a 30% reduction in invasion potential. Similarly, *C. albicans* was able to invade HeLa cells (which do not express E-cadherin) at a rate similar to invasion of cytD treated TR-146 cells.

Therefore, we have demonstrated that *C. albicans* invades epithelial cells predominantly via active penetration, whilst induced endocytosis plays a lesser, albeit significant role, during the early stages of epithelial interactions.

### Human Serum Components Protect Epithelial Cells from Invasion and Damage

Within the human host, *C. albicans* cells are permanently exposed to host molecules, which may bind to the fungal surface. Interestingly, we found that human, as well as calf sera, can reduce adhesion to oral cells. This effect may be host cell specific, as bovine serum has previously been shown to enhance *C. albicans* adhesion to endothelial cells [29]. One possible explanation for our observations is that serum factors block fungal adhesin-host ligand interactions, thereby inhibiting attachment. Since human serum had a greater inhibitory effect on attachment than bovine serum, we speculate that human serum molecules may bind to *C. albicans* adhesins or host ligands more efficiently or with greater specificity. Such interactions may also inhibit the interaction between invasins and host cell receptors required to induce endocytosis. Thus, in agreement with the study of Phan *et al.* (2005), which demonstrated inhibition of endocytosis by serum, it would appear unlikely that serum components act as bridging molecules during interactions with epithelial cells [30]. However, we only observed reduced invasiveness upon treatment with human serum, indicating that invasion-inhibitory factors are host specific and not found in bovine serum. Such human serum-specific effects have been previously reported: human but not bovine serum can inhibit apoptosis, membrane permeabilization and the release of chemokines in epithelial cells [31]; human, but not bovine, serum stimulates the expression of fibronectin by epithelial cells [32]. Therefore, it is possible that treatment with human serum triggered an epithelial reaction, which protected these cells from fungal invasion and damage. Since serum is a complex mixture of many factors, we additionally tested the influence of single extracellular host matrix (ECM) proteins on *C. albicans* adhesion and invasion capacities. However, exogenous addition of fibronectin, vitronectin, laminin or collagen did not influence invasion into oral epithelial cells. ECM proteins are highly diverse and fulfil several functions within the human host. Depending on the anatomical niche, ECM proteins are either soluble (within the plasma) or insoluble (in the extracellular matrix). They can influence cell differentiation, migration, adhesion as well as the phenotype and survival of human cells. Furthermore, ECM proteins are present as homo-, di-, or trimeric proteins and can form large protein complexes. Due to alternative splicing events, several chain variants and isofoms of the proteins are present in the host. It is possible, therefore, that the ECM proteins used in the present study were not in the correct sterical conformation, chain combination or isoform to act as bridging molecules for *C. albicans*. In contrast, addition of the cell surface protein E–cadherin led to significantly reduced fungal uptake. As epithelial-associated E–cadherin acts as a receptor for fungal invasins (Als3, Ssa1) and mediates induced endocytosis, it is possible that the addition of exogenous soluble E–cadherin saturates such fungal invasins and hence inhibits endocytosis. This observation provides further evidence that E–cadherin-binding is an important mechanism underlying induced endocytosis of *C. albicans*. In summary, we have shown that induced endocytosis and active penetration are clearly distinguishable invasion mechanisms and that viable *C. albicans* hyphae invade oral epithelial cells via both invasion routes. Active penetration represents the dominant invasion mechanism, whereas Als3-mediated induced endocytosis is critical at early time points of invasion. Moreover, we have identified the existence of an E–cadherin independent endocytic pathway. Finally, we provide compelling evidence that human serum has protective properties, inhibiting *C. albicans* adhesion to, invasion into and damage of oral epithelial cells.

## Materials and Methods

### Strains and Media

*Candida albicans* strains SC5314 [33], [34], CAI4 [33] carrying CIp10 [35], *als3*Δ [36] and *ssa1*Δ [11] strains were used in this study. All strains were maintained on YPD plates (1% peptone, 1% yeast extract, 2% glucose, 2% agar). For use in experiments, *C. albicans* cells from an overnight YPD culture were either diluted to OD_600_ = 0.2 in fresh liquid YPD medium and grown to log phase for a further 4 h at 30°C or semisynchronized for a further 24 h in liquid YPD medium at 30°C [37]. *C. albicans* cells were then harvested by centrifugation, counted with a hemacytometer and adjusted to the desired concentration in serum-free DMEM medium (containing 2 mM L-glutamine) immediately prior to the experiment.

### Epithelial Cells

The squamous carcinoma of buccal mucosa derived epithelial cell line TR-146 was obtained from Cancer Research Technology, London [38]. The HeLa cell line was commercially obtained from the Deutsche Sammlung von Mikroorganismen und Zellkulturen GmbH (DMSZ) (ACC 57) [39]. These cells were routinely cultured (passages 4 to 20) in DMEM medium supplemented with 10% FCS, 1 mM pyruvic acid and 2 mM L-glutamine (all media from Biochrom AG, Berlin, Germany), without antibiotics or antifungal agents. All cell types were maintained in a humidified incubator at 37°C in 5% CO_2_. For standard experiments, 1×10^5^ of TR-146 or HeLa cells were seeded onto acetic acid treated 15 mm diameter glass coverslips previously placed in 24-well plates and cultured for 2–3 days post-seeding.

### Invasion Assay

The number of *C. albicans* cells that invaded epithelial cells was determined using a differential staining protocol derived from Park et *al*. (2005) [40]. Briefly, epithelial cells were grown on 15 mm diameter glass coverslips for 2 days (monolayers of TR-146 and HeLa cells). To block induced endocytosis, epithelial cells were pre-treated with 0.5 µM of the microfilament inhibitor cytochalasin D (cytD) for 30 min and co-incubated throughout the whole experiment. At 0.5 µM, cytD inhibits rearrangement of the host cell actin cytoskeleton, but does not influence fungal viability, morphology or growth [9], [40]. Since cytD-treated host cells cannot endocytose killed hyphae at 3 h, we concluded that cytD blocks endocytosis over a period of at least 3 h. The monolayers were infected with 10^5^ log phase yeast cells of *C. albicans*. For determining induced endocytosis, *C. albicans* yeast cells were either grown to log phase or grown for 3 h on a plastic surface in DMEM without serum at 37°C, 5% CO_2_ and 95% humidity. Next, cells were inactivated with 100 mM thimerosal for 45 min (which kills 100% of all treated cells (data not shown)), extensively washed with water and PBS, scraped off and added to the epithelial cells. After 2 or 3 h incubation, the medium covering the cells was aspirated and monolayers were rinsed three times with PBS to remove fungal cells, which were not associated with epithelial cells. For epithelial membrane staining, the cells were incubated with Vybrant DiI cell-labeling solution (Molecular Probes, USA) 1∶20 in DMEM for 5 min in a humidified incubator at 37°C. Next, the epithelial cells were fixed with 4% paraformaldehyde. All fungal cells remaining adherent to the surface of the epithelial cells were stained for 1 h with green-fluorescent Alexa Fluor 488 conjugate of succinylated concanavalin A (Con A) (Invitrogen) (note: ConA stains only the extracellular, non-invaded fungal elements). After rinsing with PBS, epithelial cells were permeabilized in 0.5% Triton X-100 in PBS for 5–10 min. Next, entire fungal cells (*i.e.* invaded and non-invaded) were stained with calcofluor white. The coverslips were then rinsed with water, mounted inverted onto slides, and the stained cells were visualized with epifluorescence (Leica DM5500B, Leica DFC360 FX) using filter sets to detect Alexa Fluor 488, 568 and calcofluor. The percentage of invading *C. albicans* cells was determined by dividing the number of [partially] internalized cells by the total number of adherent cells. At least 50 fungal cells were counted on each coverslip and all experiments were performed in duplicates on at least two separate occasions. In the case of thimerosal-killed cells, on some occasions, fewer cells were counted as fewer dead cells remained adhered to the epithelium. To score adhesion, 25 random high powered fields per coverslip were analyzed. Images were taken with a Leica Digital Camera DFC360 FX.

To test whether invasion requires viable host cells, TR-146 cells were killed with 4% paraformaldehyde per well before infection with *C. albicans*.

In experiments designed to test the effect of extracellular matrix proteins or human serum on the endocytosis of *C. albicans* by epithelial cells, invasion assays were performed as described above except that either TR-146 cells were pre-treated with FBS-free culture medium containing 20 µg fibronectin, 500 ng vitronectin, 1 µg E-cadherin, 20 µg laminin, or 5 µg collagen per well before infection with *C. albicans* or fungal cells were pre-treated with ECMs or 40% serum 1 h before killing with thimerosal. Similar concentrations of ECM have previously been shown to interact with microbes and epithelial cells [14], [41], [42], [43], [44], [45]. The assay for TR-146 treatment was performed in DMEM with 10% serum or the indicated amount of ECMs throughout the whole experiment. Serum from at least 5 donors were pooled prior to use in experiments. Control cells were treated with water.

For the concentrations selected, none of the ECM components used affected fungal viability as determined by plating a fraction of the medium containing *C. albicans* cells and counting CFUs or by determination of hyphal length with or without inhibitor.

### Damage Assay

Epithelial cell damage caused by *C. albicans* during interaction with monolayers of TR-146 cells was determined by the release of lactate dehydrogenase (LDH) into the surrounding medium. TR-146 monolayers were grown to 95% confluency in 96 well culture plates and infected with 2×10^4^ cells in DMEM with 1% FCS. For control samples, TR-146 cells were incubated with DMEM medium only or DMEM containing 0.5% Trion X-100; additionally, *C. albicans* cells were seeded without epithelial cells. To test the influence of serum on damage, *C. albicans* cells were treated with 40% heat-inactivated or untreated human (hSerum) or fetal bovine serum (FBS). After rinsing, the fungal cells were added to monolayers of epithelial cells or untreated wild type cells were incubated with monolayers of epithelial cells in DMEM medium supplemented with 10% heat-treated or untreated hSerum or FBS. After 24 h extracellular LDH release into the medium was measured spectrophotometrically at 492 nm using the Cytotoxicity Detection Kit (LDH) from Roche Applied Science according to the manufacturer’s instructions. The percentage cytotoxicity of epithelial cells infected with *C. albicans* cells was calculated as follows: experimental LDH release minus (background cells plus background *Candida*)/mean maximal LDH release minus background cells and compared to 100% ctr. All experiments were performed in triplicates for each condition and repeated three times. For statistical analysis, *p*-values <0.05 were considered as significant.

### Transmission Electron Microscopy

For TEM, oral epithelial cells (TR-146) were grown on polystyrene plastic in DMEM with 10% FBS. After formation of a confluent layer, epithelial cells were treated for 15 min with paraformaldehyde to kill the cells or were left untreated and either thimerosal killed or viable *C. albicans* SC5314 in DMEM were added. For blocking induced endocytosis, oral epithelial cells were treated with 0.5 µM cytochalasin D as described above. After 3 h incubation non-adherent cells were removed by rinsing three times with PBS and fixed with Karnovsky fixative (7.5% Paraformaldehyde, 3.6% Glutaraldehyde, pH 7.2). Post-fixed samples (1% OsO4, 1 h) were rinsed with distilled water, block-stained with uranyl acetate (2% in distilled water), dehydrated in alcohol (stepwise 30–96%), immersed in propylenoxide and embedded in Epon (polymerized 48 h at 60 C, Serva, Heidelberg). Ultra-thin sections were stained with uranyl acetate (2% in distilled water) and lead citrate, stabilized by carbon evaporation (BAE 250, BAL TEC; Vaduz, Liechtenstein) and examined with a TEM 902 (Carl Zeiss SMT AG, Oberkochen) at 80 kV. Images were digitized using a 1K slow scan CCD – camera (Proscan, Scheuring).

### Statistical Analyses

The data were analyzed using Student’s tests to compare means. For these analyzes, *p* values of <0.05 were considered to be significant. For some experiments we chose to set the level of significance for tests at *p<*0.01.

## Supporting Information

Table S1 **Adhesion rates of** ***C. albicans*** **strains to epithelial cells**. Values represent the average percentage (with standard deviation) of adhesion at 3 h of indicated *C. albicans* strains (either viable or thimerosal-inactivated) to HeLa or TR-146 epithelial cells (either untreated, Ctrl, or treated with cytochalasin D).
(DOC)
