# Supplementary material for: Candida albicans-Epithelial Interactions: Dissecting the Roles of Active Penetration, Induced Endocytosis and Host Factors on the Infection Process
Source: PLoS One. 2012 May 14;7(5):e36952. doi: 10.1371/journal.pone.0036952 (PMC3351431; doi:10.1371/journal.pone.0036952)
Supplement: Table S1 — Adhesion rates of C. albicans strains to epithelial cells. Values represent the average percentage (with standard deviation) of adhesion at 3 h of indicated C. albicans strains (either viable or thimerosal-inactivated) to HeLa or TR-146 epithelial cells (either untreated, Ctrl, or treated with cytochalasin D). (DOC) [file pone.0036952.s001.doc]

| Strain | TR-146  Ctrl | TR-146  CytochalasinD | TR-146  Thimerosal | HeLa  Ctrl | HeLa  Thimerosal |
| --- | --- | --- | --- | --- | --- |
| Wt | 72 ± 26% | 63 ± 13% | 7 ± 7% | 76 ± 9% | 18 ± 3% |
| *als3*Δ | 3 ± 2% | 2 ± 1% | 5 ± 3% | 44 ± 4% | 5 ± 1% |
| *als3*Δ*+ALS3* | 64 ± 15% | 108 ± 62% | 14 ± 4% | 74 ± 3% | 17 ± 3% |
| *ssa1*Δ | 100 ± 19% | 67 ± 20% | 13 ± 4% | 70 ± 6% | 18 ± 2% |
| *ssa1*Δ+*SSA1* | 70 ± 16% | 69 ± 13% | 8 ± 6% | 71 ± 5% | 18 ± 4% |
